# Characterization of pre-mRNA Splicing Defects Caused by *CLCN5* and *OCRL* Mutations and Identification of Novel Variants Associated with Dent Disease

**DOI:** 10.3390/biomedicines11113082

**Published:** 2023-11-17

**Authors:** Glorián Mura-Escorche, Ana Perdomo-Ramírez, Elena Ramos-Trujillo, Carmen Jane Trujillo-Frías, Félix Claverie-Martín

**Affiliations:** 1 Unidad de Investigación, Grupo RenalTube, Hospital Universitario Nuestra Señora de Candelaria, 38010 Santa Cruz de Tenerife, Spain; glorianmuraescorche@gmail.com (G.M.-E.); atter_rad@hotmail.com (A.P.-R.); ctrufrix@gobiernodecanarias.org (C.J.T.-F.); 2 Departamento de Medicina Interna, Dermatología y Psiquiatría, Facultad de Medicina, Universidad de la Laguna, 38071 Santa Cruz de Tenerife, Spain; 3 Departamento de Medicina Física y Farmacología, Facultad de Medicina, Universidad de la Laguna, 38071 Santa Cruz de Tenerife, Spain

**Keywords:** Dent disease, *CLCN5*, *OCRL*, minigene system, bioinformatics analysis, Pre-mRNA splicing

## Abstract

Dent disease (DD) is an X-linked renal tubulopathy characterized by low-molecular-weight proteinuria, hypercalciuria, nephrocalcinosis, nephrolithiasis and progressive renal failure. Two-thirds of cases are associated with inactivating variants in the *CLCN5* gene (Dent disease 1, DD1) and a few present variants in the *OCRL* gene (Dent disease 2, DD2). The aim of the present study was to test the effect on the pre-mRNA splicing process of DD variants, described here or in the literature, and describe the clinical and genotypic features of thirteen unrelated patients with suspected DD. All patients presented tubular proteinuria, ten presented hypercalciuria and five had nephrolithiasis or nephrocalcinosis. *CLCN5* and *OCRL* genes were analyzed by Sanger sequencing. Nine patients showed variants in *CLCN5* and four in *OCRL*; eight of these were new. Bioinformatics tools were used to select fifteen variants with a potential effect on pre-mRNA splicing from our patients’ group and from the literature, and were experimentally tested using minigene assays. Results showed that three exonic missense mutations and two intronic variants affect the mRNA splicing process. Our findings widen the genotypic spectrum of DD and provide insight into the impact of variants causing DD.

## 1. Introduction

Dent disease (DD) is a rare X-linked tubulopathy that affects the function of the proximal tubule [1,2,3]. Histological studies of DD kidney biopsies have shown glomerular damage, and recent findings indicate that DD should also be considered as a podocytopathy [4]. The main characteristics of DD include low-molecular-weight proteinuria (LMWP), hypercalciuria, nephrocalcinosis, nephrolithiasis and progressive renal failure [1,2,3]. DD usually presents in children or young adults, and 30 to 80% of males affected will develop chronic kidney disease (CKD) between 30 and 50 years of age [1,2]. In addition, other manifestations indicative of dysfunction of the proximal tubule may occur, such as aminoaciduria, glucosuria, hyperphosphaturia, caliuresis and uricosuria, giving rise to a partial Fanconi syndrome [5]. A minority of patients develop rickets or osteomalacia [6,7]. In the case of female carriers, the phenotype is usually mild, rarely presenting nephrocalcinosis or chronic renal failure, probably due to the random inactivation of one of the two X chromosomes [5,8,9].

DD has been associated with alterations in two genes, both located on the X chromosome: *CLCN5* and *OCRL* [10,11]. *CLCN5* encodes the electrogenic chloride/proton exchanger ClC-5, which is mainly expressed in the kidney and participates in the endocytic reabsorption of low-molecular-weight proteins in the proximal tubular cells [12,13,14]. ClC-5 is a 746-amino-acid protein that expresses mainly in the epithelial cells of the kidney [15]. Its complex three-dimensional structure was deduced from high-resolution crystal structures of two homologous bacterial ClC exchangers (EcClC, from *E. coli*, and StClC from *S. typhimurium*). It has been suggested that ClC-5 functions as an homodimer, where each subunit serves as a pore and contains 18 α-helices (named A to R), arranged in such way that different residues come near each other to configure the Cl^−^ selectivity filter, which is formed mainly by helices D, F, N and R. α-helices B, G, H, I, P and Q form the interface between the two monomers [12,16]. Each ClC-5 monomer has a long cytoplasmic region including two cystathionine beta-synthase (CBS) domains [17,18] which are known to bind nucleotides and regulate the activity of other proteins [19,20]. It has been shown that variants located in these domains in ClC-5 are pathogenic, most of them affecting its electrical activity [16,21]. Between both CBS domains, ClC-5 also carries a PY-motif that binds WW domains of ubiquitin-ligases and modulates its retention in the plasma membrane [22]. DD caused by genetic changes in the *CLCN5* gene is known as Dent disease-1 (DD1) (OMIM #300009). 

The *OCRL* gene, previously associated with Lowe’s oculocerebrorenal syndrome, encodes an inositol polyphosphate-5-phosphatase (OCRL1), located in the Golgi apparatus and in early endosomes, that may play a role in trafficking and cellular endocytosis [23,24,25]. *OCRL* is expressed in practically all tissues, except in hematopoietic cells, [26] and through all segments of the nephron [27]. The OCRL1 protein has three conserved domains: a central inositol-5-phosphatase domain, an ASH motif binding to various Rab-GTPases and necessary for the correct targeting of OCRL1 towards the Golgi apparatus and endosomes, and a catalytically inactive Rho GAP-like domain at the C-terminus [28]. Connected through a short linker to the 5-phosphatase is a PH (pleckstrin homology) domain at the N-terminus of OCRL1 that cannot directly bind to phosphoinositide-containing liposomes [29]. DD caused by genetic changes in *OCRL* is known as Dent disease-2 (DD2) (OMIM #300555). In a retrospective analysis of phenotypes and genotypes of DD2 patients, Gianessello et al. found that truncating variants map in the PH and linker domain, while missense variants map in the inositol-5-phosphatase domain, and only occasionally in the ASH-RhoGAP module. They also observed that truncating variants located at the 5′ end of the *OCRL* gene appeared to cause the least severe phenotypes [30]. *CLCN5* variants are present in approximately 60% of patients, whereas *OCRL* variants are found in only 15% of patients. About 25% of DD patients do not harbor changes in either of these genes, and the genetic cause of these cases remains unidentified [1]. 

Understanding the effect of mutations on molecular processes is essential in order to establish genotype–phenotype correlations, which are lacking in DD. However, results of a recent study suggest that DD1 characteristics such as the risk of nephrolithiasis and progression to kidney failure are associated with the degree of remaining ClC-5 function [31]. 

It was long assumed that missense variants only changed one amino acid for another, and that synonymous variants had no effect on the protein at all. However, in the last two decades, it has become evident that presumed missense and synonymous variants can also affect the splicing of messenger RNA precursors (pre-mRNA), thus potentially having a much more severe effect on the function and expression of a protein [32,33,34]. Pre-mRNA splicing is the process by which introns are removed and exons are accurately joined together to generate mature mRNAs for the synthesis of proteins [35]. This process is regulated by splicing factors that bind sequences in the pre-mRNA, including the donor and acceptor splice sites, the branch point, the polypyrimidine track, exonic splicing enhancers (ESEs) and silencers (ESSs) and intronic splicing enhancers (ISEs) and silencers (ISSs) [36,37,38,39]. Disruption of these sequences can lead to defects in the mRNA molecules such as whole exon skipping, loss of an exon fragment or inclusion of an intron sequence, causing disease [39,40,41,42,43,44]. In fact, between 15 and 50% of all pathogenic variants have been shown to alter splice sites and splicing regulatory elements [45]. In the present study, we analyzed 13 new cases with a clinical diagnosis of DD and investigated the effect of selected variants on the splicing of the pre-mRNA.

## 2. Materials and Methods

### 2.1. Patients

Thirteen unrelated male patients diagnosed with DD and 27 of their relatives were investigated. Ten of these patients were from Spain, two were from Cuba and one was from Uruguay. Criteria for DD diagnosis were LMWP, defined by excessive urinary loss of β2-microglobulin, or total proteinuria, and at least one of the other features of DD, like hypercalciuria, defined by >4 mg/kg/d, and nephrocalcinosis/nephrolithiasis. The age for clinical diagnosis ranged from 0.8 to 19 years.

This study was approved by the Ethics Committee of Nuestra Señora de Candelaria University Hospital (Santa Cruz de Tenerife, Spain), and written informed consent for the genetic analysis was obtained from all patients and/or their parents.

### 2.2. DNA Extraction, Amplification and Sequencing Analysis

Genomic DNA was extracted from peripheral blood samples using the GenElute Blood Genomic DNA kit (Sigma-Aldrich, St. Louis, MO, USA) according to the manufacturer’s instructions. Coding exons and flanking intronic sequences of *CLCN5* and *OCRL* were amplified by polymerase chain reaction (PCR) as described previously [7,11]. PCR products were analyzed by agarose gel electrophoresis, and the fragments were purified with the NucleoSpin Gel and PCR Clean-up kit (Macherey-Nagel, Düren, Germany). DNA sequencing was performed by Macrogen Spain (Madrid, Spain). Variants were identified by comparison to the respective reference sequences (GenBank accession numbers NG_007159.3 (Transcript: ENST00000307367.2) and NG_008638.1 (Transcript: ENST00000371113.9) for *CLCN5* and *OCRL*, respectively) using the bioinformatics program Basic Local Alignment Search Tool (BLAST) (available online: https://blast.ncbi.nlm.nih.gov/Blast.cgi, accessed on 20 September 2019) and confirmed by sequencing additional independent amplification products. Several databases of genetic variants, including the Genome Aggregation Database v2.1.1 (gnomAD) (available online: https://gnomad.broadinstitute.org/, accessed on 5 June 2023) [45], 1000 Genomes Project (available online: http://www.1000genomes.org/, accessed on 5 June 2023) [46], dbSNP (available online: https://www.ncbi.nlm.nih.gov/snp/, accessed on 5 June 2023) [47] and Human Gene Mutation Database (HGMD), (Available on: http://www.hgmd.cf.ac.uk/ac/index.php, accessed on 5 June 2023) [48], were queried for the presence of the new variants identified.

### 2.3. In Silico Prediction Analysis and Criteria for Variant Selection

Missense *CLCN5* variants were selected (see selection criteria below) from the literature and from the HGMD database [48,49]. We also included *CLCN5* and *OCRL* variants identified in our new patients. These variants were analyzed using bioinformatics tools to predict their pathogenicity and their potential effects on splicing (Tables S2 and S3). Nucleotide numbering was based on the *CLCN5* and *OCRL* cDNA sequences (GeneBank accession number NM_000084.5 and NM_000276.3, respectively), with c.1 denoting the first position of the translation start codon. The criteria for selection of variants with potential effect in pre-mRNA processing were (a) proximity to splice sites (less than 70 nucleotides from the donor or acceptor splice sites) and (b) potential effect on pre-mRNA splicing predicted by at least two out of three bioinformatics tools. The following bioinformatics tools were used: MutPredSplice v1.3.2 (available online: http://www.mutdb.org/mutpredsplice, accessed on 20 October 2019) [50], splicing-based analysis of variants (SPANR) (available online: http://tools.genes.toronto.edu/, accessed on 15 November 2019) [51] and Human Splicing Finder (HSF) v3.1 [52] (available online: https://hsf.genomnis.com/, accessed on 10 August 2023). In addition, we used other bioinformatics tools like Splice Site Prediction by Neural Network v0.9 (NNSplice) (available online: https://www.fruitfly.org/seq_tools/splice.html, accessed on 20 July 2020) [53], CADD-splice v.1.6 (Combined Annotation Dependent Depletion) (available online: https://cadd.gs.washington.edu/, accessed on 20 February 2022) [54] and SpliceAI v1.3.1 (available online: https://spliceailookup.broadinstitute.org/, accessed on 2 November 2023) [55].

The effect of amino acid substitutions on the ClC-5 and OCRL1 proteins was predicted using the following bioinformatics tools: PolyPhen-2 (Available online: http://genetics.bwh.harvard.edu/pph2/, accessed on 22 June 2022) [56], SIFT v6.2.1 (available online: https://sift.bii.a-star.edu.sg/, accessed on 22 June 2022) [57] and MutPred2 (available online: http://mutpred.mutdb.org/, accessed on 30 November 2022) [58] to predict whether an amino acid substitution in a protein would have a phenotypic effect. Multiple sequence alignment of proteins was performed using Clustal Omega v1.2.4 (available online: https://www.ebi.ac.uk/Tools/msa/clustalo/, accessed on 30 January 2023) [59]. The pathogenicity of variants was also determined using the VarSome suite v11.5 (available online: https://varsome.com/, accessed on 15 December 2022) [60]. According to the American College of Medical Genetics and Genomics (ACMG) recommendations, variants were classified into five categories, such as pathogenic, likely pathogenic, uncertain significance, likely benign and benign [61]. 

### 2.4. Amplification of CLCN5 and OCRL Genomic Fragments and Construction of Minigenes

The effect of *CLCN5* and *OCRL* variants on pre-mRNA splicing was evaluated using a minigene system and reverse transcription-PCR (RT-PCR) analysis. For the construction of *CLCN5* minigenes, four fragments containing exons 3, 7, 9 and 10–11 and their flanking intronic sequences were cloned separately in the pET01 expression vector (MoBiTec, Göttingen, Germany) (intron 2 (172 bp)-exon 3 (100 bp)-intron 3 (85 bp) (pET01ex3-WT); intron 6 (196 bp)-exon 7 (81 bp)-intron 7 (140 bp) (pET01ex7-WT); intron 8 (104 bp)-exon 9 (187 bp)-intron 9- (133 bp) (pET01ex9-WT); and intron 9 (162 bp)-exon 10 (399 bp)-intron 10 (155 bp)-exon 11 (217 bp)-intron 11-(149) (pET01ex10-11). These fragments were amplified by PCR from genomic DNA extracted from patients and/or controls (Table S1). For *OCRL* minigenes, two fragments containing exons 11–12 and 15 and flanking intronic sequences (intron 10 (84 bp)-exon 11 (117 bp)-intron 11 (98 bp)-exon 12 (188 bp)-intron 12 (84 bp) (pET01ex11-12WT) and intron 14 (24 bp)-exon 15 (336 bp)-intron 15 (40 bp) (pET01ex15WT)) were amplified from the genomic DNA of the patients and a healthy control. 

Primers were designed using Primer3 v0.4.0 (Available online: https://primer3.ut.ee/, accessed on 20 October 2019) and SnapGene software v5.0.4 (Available online: www.snapgene.com, accessed on 25 October 2019). Primers contained sequences encoding restriction sites for *XhoI, XbaI* and *BamHI* at their 5′ ends. PCR reactions were carried out using a Kapa Taq PCR kit (Kapa Biosystems—Hoffman-La Roche, Wilmington, MA, USA). After digestion with restriction enzymes *XhoI, XbaI* or *BamHI* (Thermo Fisher Scientific, Waltham, MA, USA), PCR products were cloned using T4 DNA ligase (Kapa Biosystems), according to the manufacturer’s instructions, into the pET01 previously digested with the respective restriction enzymes. Ligation products were transformed into XL1 Blue competent cells by heat-shock and grown in Luria-Bertani Broth (LB) agar plates supplemented with ampicillin. Colonies carrying recombinant plasmids were grown overnight in LB medium with ampicillin at 37 °C. Plasmid DNA extraction was carried out with a NucleoSpin Plasmid EasyPure kit (Macherey-Nagel, Düren, Germany), and recombinant plasmids were analyzed by sequencing (Macrogen Spain, Madrid, Spain).

### 2.5. Site-Direct Mutagenesis

*CLCN5* mutations were introduced in the respective minigenes using the QuickChange^®^ Lightning Site-Directed Mutagenesis Kit (Agilent Technologies, Santa Clara, CA, USA) following the manufacturer’s recommendations. Reaction products were transformed into XL10-Gold ultracompetent cells. Primers for mutagenesis were designed using the bioinformatics tool QuickChange^®^ Primer Design Program (available online: https://www.agilent.com/store/primerDesignProgram.jsp, accessed on 20 October 2019) according to the guidelines described in the QuickChange^®^ commercial kit (Table S1). To confirm the presence of the desired mutation, all constructs were analyzed by directed sequencing using the same primers used for the amplification of each fragment.

### 2.6. Cell Culture, Transient Transfection and RT-PCR Assay

COS7 and HEK293T cells were cultured in Dulbecco’s Modified Eagle Medium (DMEM, Sigma-Aldrich) with high glucose (4.5 g/L), supplemented with 10% foetal bovine serum and 1% penicillin/streptomycin and incubated at 37 °C and 5% CO_2_ in a humidified incubator. Minigenes were transfected using JetPRIME (Polyplus Transfection, Illkirch, France), according to the manufacturer’s instructions. After 24h, RNA was extracted and purified using the NucleoSpin RNA mini kit (Macherey-Nagel, Düren, Germany) and quantified with Nanodrop Lite (Thermo Fisher Scientific). cDNA synthesis was performed with an iScript cDNA synthesis kit (Bio-Rad, Hercules, CA, USA) and second strands were amplified by PCR with a Kapa Taq PCR kit (Kapa Biosystems-Hoffman-La Roche) and DreamTaq polymerase (Thermo Scientific, Thermo Fisher Scientific Inc.) using primers ETprim02 and ETprim03 (MoBiTech, Göttingen, Germany). Products were analyzed by agarose gel electrophoresis with the molecular weight marker SiZer-100bp DNA Marker (IntRon Biotechnology DR, Gyeonggi, Republic of Korea) and sequenced as mentioned before. The exact size of each product was determined from the DNA sequences, which were compared to the reference *CLCN5* or *OCRL* sequences (GenBank accession number NC_000023.10 and NM_000084.5 or NC_000023.11 and NM_000276.3, respectively).

## 3. Results

### 3.1. Identification of Novel CLCN5 and OCRL Variants in Patients Diagnosed with Dent Disease

Clinical data of patients included in this study and the variants identified are summarized in Table 1. All patients had LMWP, ten had hypercalciuria, and five presented nephrolithiasis or nephrocalcinosis. Patient P422 also showed other symptoms as hypotonia, attention-deficit disorder and growth hormone deficit. P854 manifested hyperaminoaciduria. Patient P508 showed developmental delay and congenital cataracts. 

**Figure 1:**
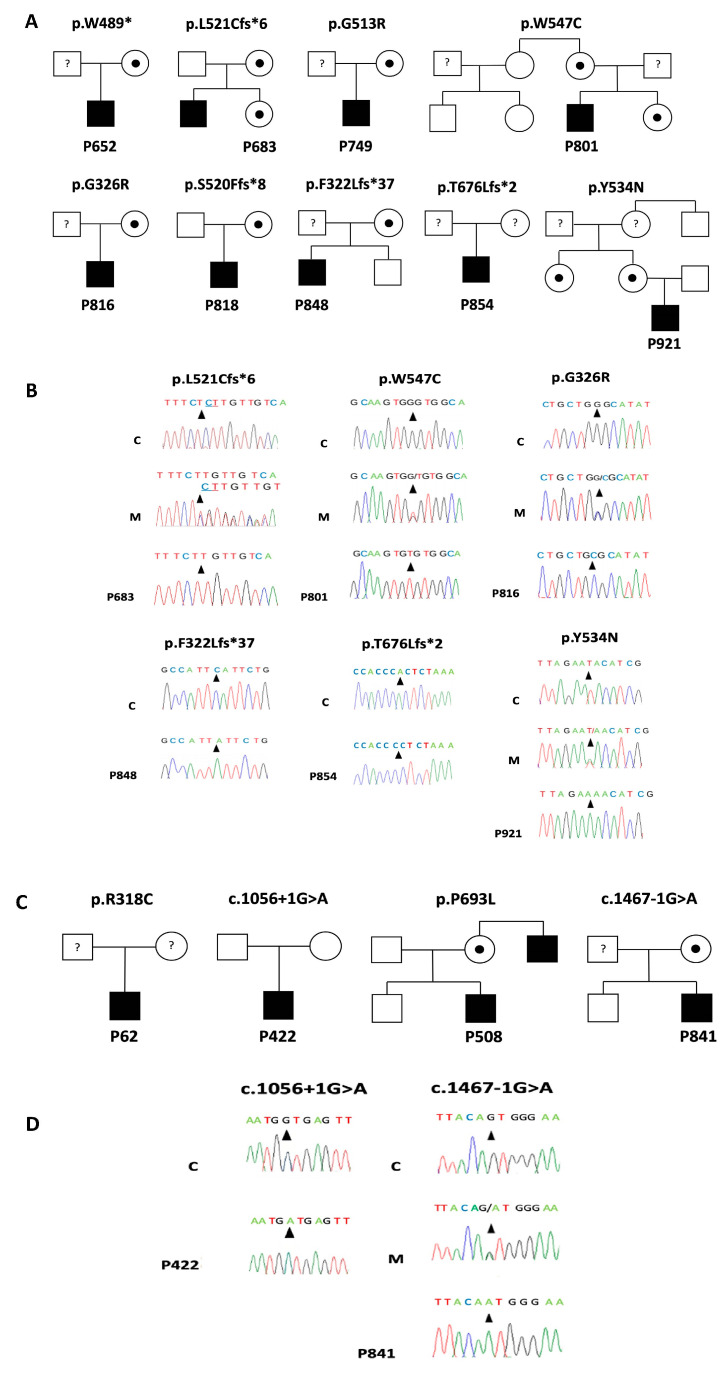
Segregation and electropherograms of the detected *CLCN5* and *OCRL* variants. (**A**) Pedigrees of families with *CLCN5* variants; (**B**) Electropherograms showing the new *CLCN5* variants; (**C**) Pedigrees of families with *OCRL* variants; (**D**) Electropherograms showing the new O*CRL* variants. Circles with a dot in the center indicate female carriers; open circles are unaffected females; filled squares are affected males; open squares are unaffected males; question marks inside circles and squares indicate unanalyzed individuals. The arrowheads indicate the nucleotide position affected; M = Heterozygous mother; C = Normal controls.

Sequence analysis revealed nine *CLCN5* variants (four missense, four frameshift and one nonsense variant) (Table 1) (Figure 1), six of which were not present in genomic variant databases (HGMD, dbSNP, gnomAD and 1000 Genomes Project) (c.1641G>T; p.(W547C), c.976G>C; p.(G326R), c.1600T>A; p.(Y534N), c.2026delA; p.(T676Lfs*2), c.1560_1561delTC; p.(L521Cfs*6) and c.966delC; p.(F322Lfs*37)). Four variants were found in the *OCRL* gene (two missense and two intronic variants), two of which were not found in the genomic variant databases mentioned above (c.1056+1G>A and c.1467-1G>A). Novel variants were submitted to the ClinVar database and were included with the following accession numbers: VCV000930215.1 (c.1641G>T); VCV001210259.1 (c.976G>C), VCV000973833.1 (c.1600T>A), VCV000932946.1 (c.2026delA), VCV000932943.1 (c.1560_1561delTC), VCV000932948.1 (c.966delC), VCV000932947.1 (c.2078C>T), VCV002506950.1 (c.1056+1G>A) and VCV002504612.1 (c.1467-1G>A). In ten families, we showed that the patients’ mothers were carriers of the respective *CLCN5* and *OCRL* variant (Figure 1). In the other families, blood samples of the parents were not available for genetic analysis.

**Figure 2:**
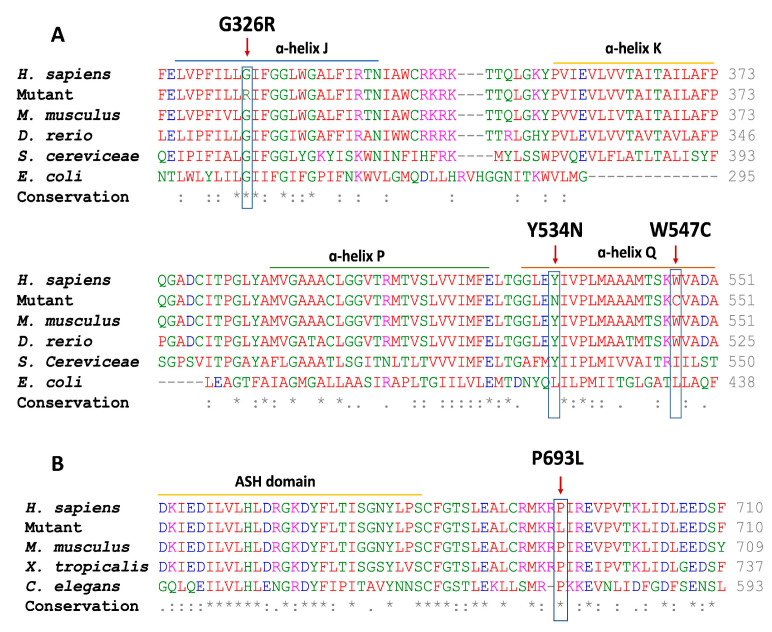
Multiple alignments of ClC-5 (**A**) and OCRL1 (**B**) protein sequences with a subset of vertebrate orthologs. The vertical arrow indicates the position of the altered amino acid residue. Residues conserved at this position are remarked with a rectangle. The α-helices J, K, P and Q of ClC-5 and the ASH domain of the OCRL1 protein are indicated. An asterisk indicates positions that have a single, fully conserved residue. A colon denotes conservation between groups of strongly similar properties. A period indicates conservation between groups of weakly similar properties. No symbol means no conservation.

The three novel *CLCN5* missense variants, c.1641G>T; p.(W547C), c.976G>C; p.(G326R), and c1600T>A; p.(Y534N), affect residues conserved through evolution and are predicted to affect protein function (Figure 2). VarSome analysis classified variant p.(G326R) as of uncertain significance according to the pathogenicity criteria established by the ACMG (Table S2), while variants p.(Y534N) and p.(W547C) were classified as likely pathogenic (Table S2). Variant p.(G326R) affects an amino acid residue located in α-helix J of the ClC-5 protein, whereas p.(Y534N) and p.(W547C) disturb residues located in α-helix Q (Figure 2). Analysis of c.976G>C; p.(G326R) and c.1600T>A; p.(Y534N) with HSF, SPARN and SpliceAI indicated that these changes have no impact on the pre-mRNA splicing process (Table S2). According to CADD, splice variant p.(W547C) seems to affect pre-mRNA splicing, and HSF predicts that this variant generates a cryptic donor site (Table S2). The other three novel *CLCN5* variants, p.(L521Cfs*6), p.(F322Lfs*37) and p.(T676Lfs*2), are single nucleotide deletions that change the open reading frame and result in the generation of premature stop codons after 5, 36 and 1 amino acids, respectively (Figure 1B). VarSome classified these frameshift variants as pathogenic (Table S2). These variants are located in α-helix K, α-helix J and residues located between the CBS-1 and CBS-2 domains, respectively.

*OCRL* variant p.(P693L) is located between the ASH and the RhoGap homology domains in the OCRL1 protein, disturbing a residue conserved through evolution and probably affecting protein function (Figure 2). Analysis with MutPredSplice, SPARN and HSF showed no potential effect on pre-mRNA processing (Table S2). VarSome ACMG classified the p.(P693L) variant as of uncertain significance (Table S2). *OCRL* variants c.1056+1G>A and c.1467-1G>A are both splice-site mutations. HSF analysis predicts the inactivation of the donor splice site of intron 11 and the inactivation of the acceptor splice site of intron 14, respectively, matching with SpliceAI predictions (Table S2). In both cases, SPARN analysis predicts a decreased expression of transcripts carrying the corresponding exon (Table S2).

### 3.2. Selection of CLCN5 Missense Variant from Databases for Their Potential Effect on pre-mRNA Splicing

We selected 63 *CLCN5* missense variants from the literature [49] that were located less than 70 nucleotides from the donor or acceptor splice sites (Table S3). From these, only 12 variants showed potential effects on pre-mRNA splicing with at least two of the three bioinformatics tools described in Materials and Methods (Table S3). The selected variants were: c.193G>A; p.(G65R) in exon 3, c.731C>T; p.(S244L), c.781G>A; p.(G261R) and c.800A>C; p.(E267A) in exon 7, c.1384G>A; p.(G462S), c.1511T>A; p.(M504K), c.1516G>A; p.(G506R), c.1517G>A; p.(G506E) and c.1534G>C; p.(G512R) in exon 9, c.1535G>A; p.(G512D), c.1537G>A; p.(G513R) and c.1639T>C; p.(W547R) in exon 10. Analysis of these variants with bioinformatics tools SIFT, PolyPhen2 and MutPred2 predict amino acid substitutions that affect the function of the ClC-5 protein (Table S4). According to VarSome, these changes are likely pathogenic, except p.(G65R), which was classified as of uncertain significance. Predictions at the mRNA level for these variants included the generation of new donor or acceptor splice sites, the inactivation of a donor splice site and the generation or inactivation of ESS or ESE sites (Table S4).

### 3.3. Functional Analysis of Variants

#### 3.3.1. Minigene Analysis of *CLCN5* Variants

**Figure 3:**
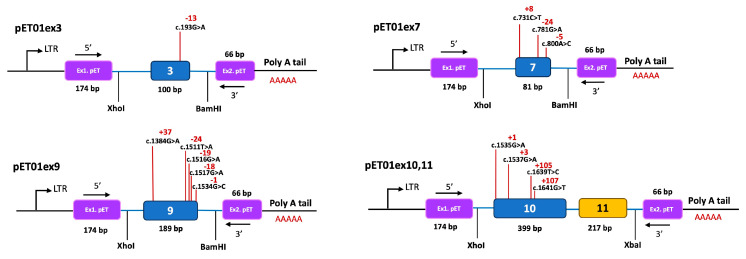
Schematic representation of minigene constructions. *CLCN5* exons are flanked by pET01 exons 1 (5′ end) and 2 (3′ end) followed by a polyadenylation site (poly A tail). Red lines indicate the locations of mutations introduced by site-direct mutagenesis. The sizes of *CLCN5* and pET01 exons are indicated. Numbers in red indicate the positions of mutations with respect to acceptor (+) or donor (-) sites. Restriction sites for *XhoI, BamHI* and *XbaI* are also shown. LTR, long terminal repeat promoter of the Rous Sarcoma Virus.

**Figure 4:**
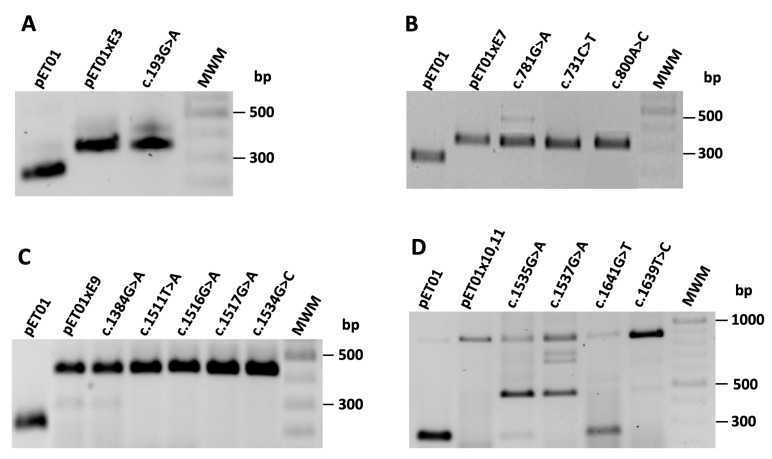
Representative agarose gels showing the results of the RT-PCR analysis of spliced transcripts expressed from *CLCN5* minigenes containing WT and mutant exons. None of the mutations located in exons 3, 7 and 9 showed altered mRNA products (**A**–**C**). (**D**) Mutations in exons 10 (c.1535G>A, c.1537G>A and c.1641G>T) generated mRNA products of the same size as the WT together with altered products.

We tested the effect on pre-mRNA splicing of the twelve variants selected using a minigene system. In this analysis, we also included presumed missense variant c.1641G>T; p(W547C), identified in exon 10 of one of our patients, which was predicted to affect protein function and pre-mRNA splicing (Table S2). Figure 3 shows the organization of each construction. We observed RT-PCR products with the expected size in each control construction carrying the WT sequences (Figure 4). Only three of the twelve variants analyzed (c.1535G>A, c.1537G>A and c.1641G>T) showed RT-PCR products different from the WT (Figure 4). 

##### Variants c.1535G>A, c.1537G>A and c.1641G>T Alter pre-mRNA Splicing of *CLCN5*

**Figure 5:**
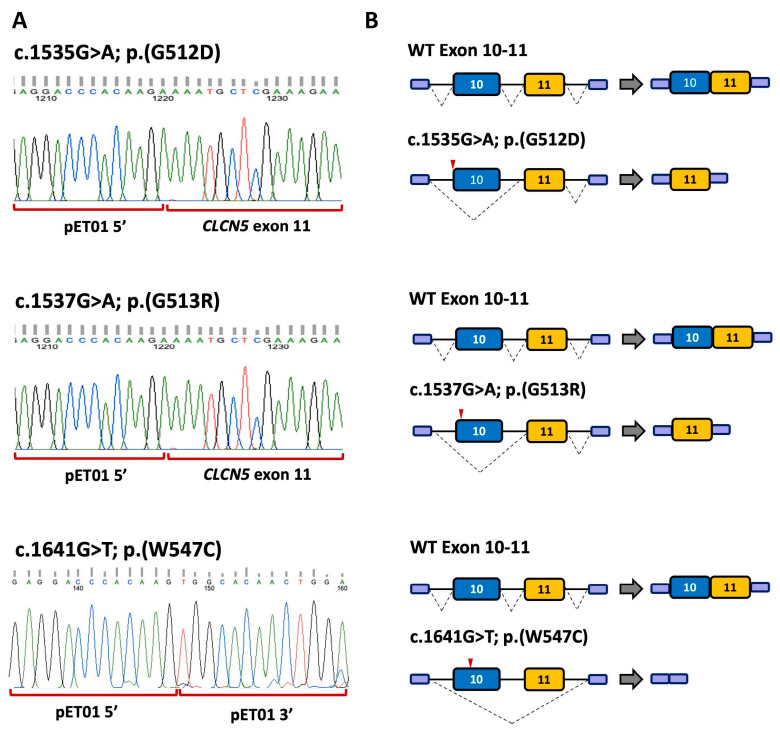
(**A**) DNA sequencing of the altered RT-PCR products from exon 10–11 constructions with mutations c.1535G>A; p.(G512D), c.1537G>A; p.(G513R) and c.1541G>T; p.(W547C) (**B**) Schematic representation of pre-mRNA splicing in WT and mutant minigenes of exon 10-11 construction. Red arrowheads indicate the location of the variant.

*CLCN5* variant c.1535G>A; p.(G512D) (Table S3 and Figure 3) changes the first nucleotide of exon 10 (tgcag**G**TGGGG; the nucleotide affected by the variants appears in bold letter, and the AG motif of the acceptor splice site in intron 9 is underlined. Intronic and exonic sequences are in small and capital letters, respectively, throughout the text). MutPredSplice and CADD-splice predictions indicated that c.1535G>A alters the acceptor splice site (Table S4). However, analysis with NNsplice showed that the acceptor splice site score changes very slightly (0.99 to 0.98), and SpliceAI does not predict any changes (Table S4). To investigate the effect of this variant, we created a minigene containing exons 10 and 11 and their flanking intronic sequences (pET01ex10-11) (Figure 3). The WT and mutant minigenes were transfected separately into HEK293T and COS7 cells, and RT-PCR analysis was performed. The results revealed a different band pattern in the electrophoresis in the WT and mutant minigene. The WT construction generated the expected splicing product with a size of 850 bp, whereas the mutant minigene generated two splicing products: a faint band of 850 bp, consistent with the WT transcript; and a band of approximately 450 bp (Figure 4D). Direct sequencing showed that the smaller product lacked exon 10 and corresponded to the pET01 exons 5′ and 3′ (Figure 5). Analysis with the HSF tool showed that c.1535G>A creates several overlapping splicing silencer motifs, including one Sironi motif 1 (gcag*A*TGG), two Sironi motif 2 (ag*A*TGGG and g*A*TGGGG) and one hnRNPA1 binding site (g*A*TGGG) (Figure S1, Table S5). c.1535G>A also generates two FAS-hex3 hexamers g*A*TGGG and *A*TGGGG [65]. The binding of splicing repressors to these sites could inhibit the recognition of the acceptor splice site by the splicing machinery, causing exon 10 skipping. 

Variant c.1537G>A; p.(G513R), identified in one of our patients (Table 1 and Table S2), is located at position +3 of exon 10 (tgcagGT**A**GGG). Analysis with MutPredSplice, CADD-splice and SpliceAI suggested that it could alter *CLCN5* pre-mRNA splicing (Tables S3 and S4). NNSplice analysis showed the same score for the acceptor splice site with and without the mutation (0.99). To investigate the consequences of c.1537G>A in pre-mRNA splicing, we used the minigene containing exons 10 and 11 (Figure 3). The WT and mutant minigenes were transfected separately into HEK293T and COS7 cells, and the mRNA products were examined by RT-PCR. Direct sequencing showed that the mutant minigene generated a 450 bp product corresponding to the skipping of exon 10 and a band of approximately 850 bp corresponding to the WT product (Figure 4D and Figure 5). Two additional bands of approximately 700 bp and 750 bp were observed (Figure 4D), but we were unable to separate them for sequence analysis. HSF showed the presence in the mutant sequence of overlapping ESS motifs, including a Sironi motif 2 (GT*A*GGGT) and an hnRNPA1 binding site (T*A*GGGTG) (Figure S1 and Table S5). Additionally, this region contains three overlapping FAS-hex3 hexamers (GGT*A*GG, T*A*GGGT and *A*GGGTG) not present in the WT sequence [65]. Binding of splicing repressors to these sites could explain exon 10 skipping in our minigene system.

Variant c.1641G>T; p.(W547C), also found in one of our patients, is located in position +107 from the 5′ end of exon 10 (Table S2). Analysis of the mutant sequence with CADD-splice suggested an effect on pre-mRNA splicing, but SpliceAI predicted no changes in the splice sites (Table S2). As expected, this variant did not change the NNSplice score of the WT acceptor splice site (0.99). Analysis with the HSF tool identified the presence of three Sironi motifs 2 (AGTG*T*GT, TG*T*GTGG and *T*GTGGCA) (Figure S1 and Table S5). Overlapping these motifs is a FAS-hex3 ESS hexamer (G*T*GTGG) [66]. RT-PCR analysis of the mutant minigene and direct sequencing revealed one band corresponding to the WT transcript and a smaller band of 240 bp in size, corresponding to the junction of the two pET01 exons (Figure 4D). Therefore, variant c.1641G>T causes the skipping of exons 10 and 11 in our minigene system (Figure 5), probably by the binding of a splicing repressor to the ESS motifs. Interestingly, HSF analysis of variant c.1639T>C; p.(W547R), located in position +105 from the acceptor site and only two nucleotides apart from c.1641G>T; p.(W547C), did not predict the generation of any ESS and did not show an aberrant pre-mRNA splicing in our minigene system (Table S4, Figure 4D).

##### Variants in Exons 3, 7 and 9 of *CLCN5* Did Not Alter pre-mRNA Splicing

Using the minigene assay, we also studied one variant in exon 3, c.193G>A; p.(G65R), located at position −13 with respect to the donor site of intron 3; three variants in exon 7, c.731C>T; p.(S244L), located at position +8 with respect to the acceptor site of intron 6, c.781G>A; p.(G261R), located at position −24 with respect to the donor splice site of intron 7 and c.800A>C; p.(E267A), located at position −5 with respect to the donor site of intron 7; and five variants in exon 9, c.1384G>A; p.(G462S), located at position +37 with respect to the acceptor splice site of intron 8, c.1511T>A; p.(M504K), located at position -24 with respect to the donor splice site of intron 9, c.1516G>A; p.(G506R), located at position −19 with respect to the donor splice site of intron 9, c.1517G>A; p.(G506E), located at position −18 with respect to the donor splice site of intron 9, and c.1534G>C; p.(G512R), located at position −1 with respect to the donor splice site of intron 9 (Figure 3). Bioinformatics analysis of these variants suggested potential alterations in pre-mRNA splicing (Table S3). Variant c.193G>A; p.(G65R) generated a new acceptor site in exon 3 according to HSF (Table S3), but SpliceAI predicted no changes in the splice sites (Table S4). The three variants located in exon 7 predicted the generation of ESSs and the abolition of ESEs according to HSF, a reduction in the number of transcripts according to SPANR, and a donor site gain in variant c.781G>A; (p.G261R) according to SpliceAI (Table S3 and S4). According to MutPredSplice and CADD-Splice, exon 9 variants c.1384G>A; p.(G462S), c.1511T>A; p.(M504K), c.1516G>A; p.(G506R) and c.1534G>C; p.(G512R) disrupted pre-mRNA splicing (Tables S2 and S4). Variants c.1517G>A; p.(G506E), c.1384G>A; p.(G462S) and c.1511T>A; p.(M504K) were predicted by HSF to generate ESSs (Table S3 and S4). According to SpliceAI, none of them alter the splice sites (Table S4). However, the results of the minigene analysis and sequencing showed that none of these variants affected pre-mRNA splicing (Figure 4).

#### 3.3.2. Minigene Analysis of OCRL Variants

**Figure 6:**
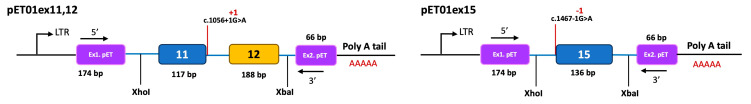
Schematic representation of minigenes constructed with expression vector pET01 and *OCRL* WT sequences. The constructions are flanked by exon 1 of pET01 in the 5′ end and by exon 2 of pET01 in the 3′ end, followed by a polyadenylation site (poly A tail). The location of mutations introduced by side-direct mutagenesis is indicated. The size of *OCRL* and pET01 5′ and 3′ exons are indicated. Numbers in red indicate the localization of mutations respect to donor (+) or acceptor (-) intronic sites. Restriction sites for *XhoI* and *XbaI* are also shown. LTR, long terminal repeat promoter of the Rous Sarcoma Virus.

Two *OCRL* intronic variants, c.1056+1G>A and c.1467-1G>A, were identified in patients from our DD cohort. Both variants affect one nucleotide at the canonical splice site of the respective donor or acceptor sites (Figure 6). Bioinformatics analyses predicted pre-mRNA alterations for both variants (Table S2).

##### *OCRL* Variant c.1056+1G>A Results in the Skipping of Exon 11

**Figure 7:**
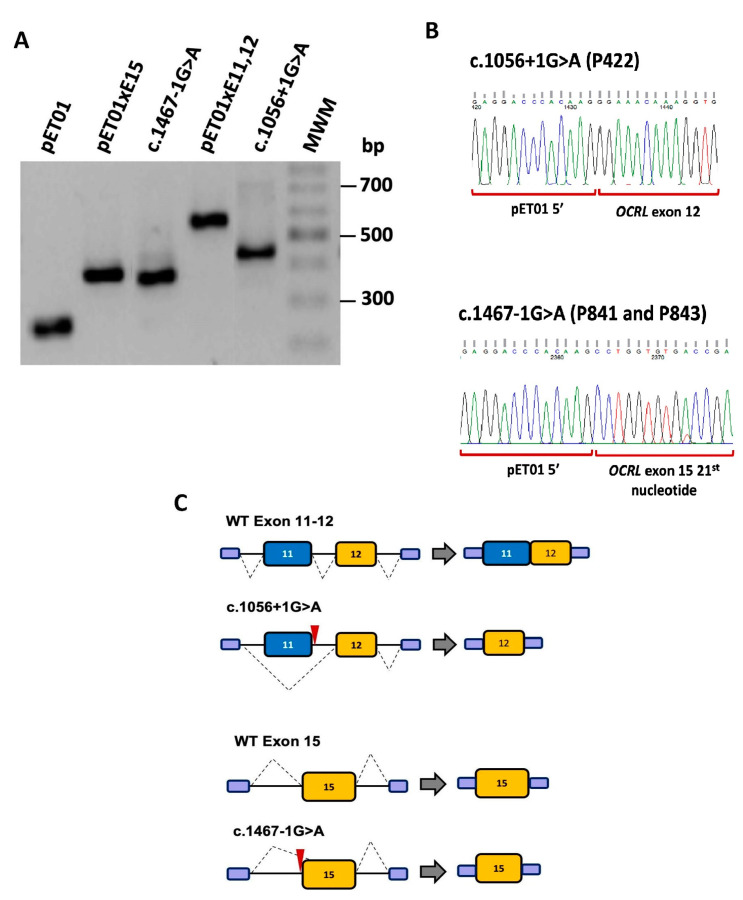
(**A**) Representative agarose gel showing the results of the RT-PCR analysis of spliced transcripts expressed from *OCRL* minigenes containing WT and mutant exons. (**B**) DNA sequencing of the altered RT-PCR products from exon 11–12 and exon 15 constructions with mutations c.1056+1G>A and c.1467-1G>A. (**C**) Schematic representation of pre-mRNA splicing in WT and mutant constructions. Exon 11 and the first 20 nucleotides of exon 15 are missing due to mutations c.1056+1G>A and c.1467-1G>A, respectively. Red arrowheads indicate the location of the variant.

Bioinformatics analysis with NNSplice and HSF predicted that variant c.1056+1G>A inactivates the canonical donor splice site dinucleotide (GU) of intron 11 (Figure 6, Table S2). According to NNSplice, the score of the WT donor splice site of intron 11 is 0.99, while the mutant score goes down 0.00. To determine the effect of this variant, we created minigenes containing WT and mutant sequences of exons 11 and 12. The WT and mutant minigenes were transfected separately into COS7 and HEK293T cells. RT-PCR results showed that the WT minigene produced a band of 545 bp corresponding to exons 11 and 12, and the mutant construction produced a unique smaller band of 420 bp consistent with skipping of exon 11 (Figure 7A,C). Direct sequencing analysis of the RT-PCR products confirmed these results (Figure 7B). 

##### *OCRL* Variant c.1467-1G>A Results in Incorporation of a Truncated Exon 15 in the mRNA

Variant c.1467-1G>A was predicted by the HSF tool to inactivate the conserved dinucleotide (AG) at the canonical acceptor splice site of intron 14 (Figure 6, Table S2). Accordingly, the NNsplice scores for the WT and mutant sites were 0.92 and 0.00, respectively. To investigate the effect of this variant, we created a minigene harbouring exon 15 and its flanking intronic sequences. The results of the RT-PCR analysis showed a unique product of 350 bp in the mutant minigene and a larger band of 370 bp in the WT minigene (Figure 7A). Sequencing analysis of these products confirmed that the smaller fragment matches the incorporation of a truncated exon 15 missing 20 nucleotides from the 5′ end, and that the product from the WT minigene corresponds to the correctly spliced exons (Figure 7B,C). HSF analysis predicted the presence of a cryptic acceptor splice site located 18 nucleotides downstream from the beginning of exon 15 (GTTCCAGCCTGG). The use of this site by the splicing machinery would explain the incorporation of the truncated exon 15 in the mRNA. 

## 4. Discussion

We report the identification of six new exonic variants in *CLCN5* associated with DD1 (three presumed missense variants and three small deletions) and two novel canonical splice site variants in *OCRL* associated with DD2 and the functional effects on pre-mRNA splicing of three of these variants and 12 variants previously identified in DD patients. Bioinformatics predictions of the novel variants identified in our study indicate that they are all pathogenic (Table S2). *CLCN5* missense variants p.(W547C), p.(Y534N) and p.(G326R) and *OCRL* missense variant p.(P693L) affect conserved amino acid residues of the corresponding proteins. Two other variants in codon 547, p.(W547R) and p.(W547G) have been previously identified in patients with DD1 [67,68], indicating that this tryptophan residue is important for the proper functioning of the ClC-5 protein. On the other hand, we have previously shown that variant p.(W547G) increases the expression of *CLCN5* mRNA isoform lacking exons 10 and 11 in the patient’s lymphocytes [68]. In the present study, we showed that variant p.(W547C) also has a similar effect (discussed below). Furthermore, expression studies in oocytes have shown that p.(W547G) reduces significantly ClC-5 currents [69]. These data suggest that variant p.(W547C) is pathogenic. Nevertheless, further studies are needed to assess the effect of the new missense variants identified in our study on the activity of the ClC-5 protein. The three small *CLCN5* deletions identified result in frameshifts and generation of premature stop codons, which could lead to complete loss of protein expression due to nonsense-mediated mRNA decay (NMD) [70].

*OCRL* missense variant p.(R318C), identified in one of our patients, affects the 5-phosphatase domain of OCRL1. This variant is a recurrent variant that has been described in at least 13 unrelated families with DD2 from different countries. Another variant affecting the same codon, p.(R318H), has been described in six unrelated DD2 families. Codon 318 is considered as a mutational hot spot in the *OCRL* gene [30]. The majority of reported DD2 and Lowe syndrome missense variants map in the 5-phosphatase domain of the OCRL1 protein [30,71]. Gianesello and col. studied the distribution of DD2 causing variants in relation to extra-renal symptoms and found that patients with variants in the 5-phosphatase domain presented mainly with muscular involvement, central nervous system (CNS) symptoms and rarely with ocular defects [30]. The majority of patients carrying variant p.(R318C) did not present any extra-renal symptoms; only seven presented mild CNS and/or muscular alterations, and only two presented ocular defects [30]. Accordingly, our patient carrying p.(R318C) presented renal symptoms such as LMWP, hypercalciuria and nephrocalcinosis, and did not show any extra-renal alteration. On the other hand, missense variant p.(P693L) is one of the few DD2 causing variants that is located in the ASH-RhoGap module [30,62]. Ocular symptoms are very rare in DD2 cases, and, when present, they are more frequently related to variants in the ASH and Rho-GAP domains. In addition, one third of variants located in this region cause CNS alterations [30]. In accordance with these observations, our patient carrying variant p.(P693L) showed congenital cataracts and developmental delay, together with renal symptoms like LMWP and hypercalciuria. 

Alteration of the pre-mRNA splicing process by intronic or exonic variants is a well-established cause of disease [32,33,34]. More recently, it has become evident that exonic variants that affect this process are more prevalent than previously predicted [72]. In order to improve the genetic diagnosis and the design of new therapeutic strategies for hereditary diseases, it is necessary to evaluate the biological and clinical consequences of presumed splicing variants [73]. Bioinformatics tools that predict splicing defects can be used initially as supplementary evidence for genetic diagnosis. Nevertheless, functional assays using RNA from patients or minigenes are required to evaluate the pathogenicity of a gene variant [61]. A recent functional study of intronic *CLCN5* variants, located near the exons’ ends (3 to 17 nucleotides), using a minigene assay, has shown that five of these variants produce altered mRNAs and concluded that they are pathogenic [74]. For instance, variant c.393+4A>G, close to the donor splice site of intron 4, results in exon 4 skipping, and variant c.517-3C>A, close to the acceptor splice site of intron 5, induces both exon 6 skipping and partial deletions of exon 6. These studies are also necessary for variants that affect canonical dinucleotide of the splice sites, which are usually considered pathogenic since they result in complete absence of the protein due to NMD of the altered transcript [70]. RNA samples from patients’ tissues are generally problematic to obtain, and minigene assays are a practical alternative [75]. Therefore, we used here the minigene system we have successfully employed in previous studies [76,77,78,79,80].

*CLCN5* exonic variants c.1535G>A [67] and c.1537G>A [68], located close to the 5′ end of exon 10 (positions +1 and +3, respectively), do not change the NNSplice score of the acceptor splice site, however, we found that both variants generate different overlapping ESS motifs. ESSs seem to function by interacting with splicing repressors, which are RNA-binding proteins of the heterogeneous nuclear ribonucleoprotein (hnRNP) family (such as hnRNPA1) [36,81]. These ribonucleoproteins contain RNA-binding domains for binding to the nascent pre-mRNA and an inhibitory domain involved in protein–protein interactions. It has been proposed that hnRNPs cover a region in the exon and compete with splicing enhancers, blocking their binding [81,82]. We suggest that binding of repressors to the ESSs generated by variants c.1535G>A and c.1537G>A could prevent the recognition by the splicing machinery of the canonical acceptor site, inducing the skipping of exon 10. Interestingly, variant c.1641G>T, located 107 nucleotides from the 5′ end of exon 10, also generates several overlapping ESS motifs, but it results in skipping of not only exon 10 but also exon 11. We have shown before, using RNA from a patient’s lymphocytes, that variant c.1639T>G, which is located in the same codon as c.1641G>T, increases the expression of the mRNA isoform lacking exons 10 and 11 [68]. This variant c.1639T>G also generates several overlapping ESSs in the same region (Figure S1). Tosetto and col. have previously identified a different variant but in the same nucleotide, c.1639T>C, in another DD1 patient [67]. Remarkably, this variant does not generate ESSs and does not alter pre-mRNA splicing (Figure 4D). From these results, we conclude that binding of repressor(s) to ESSs in this region could displace positive regulatory proteins or other spicing factors, resulting in the skipping of exons 10 and 11. Further studies will be needed to understand the mechanism that leads to the simultaneous skipping of both exons induced by variants c.1639T>G and c.1641G>T. 

The results of our studies showed that minigenes containing variants c.1535G>A; p.(G512D), c.1537G>A; p.(G513R) and c.1641G>T; p.(W547C) produce a residual amount of transcripts containing exons 10 and 11. Therefore, we suggest that these exonic variants act at the protein and RNA levels, causing both altered pre-mRNA splicing and the corresponding amino acid change. The same assumption would apply to the previously studied variant c.1639T>G; p.(W547G) [68]. Protein expression and electrophysiological studies have shown that this variant yields reduced surface expression of the ClC-5 mutant protein and drastically reduced currents at the plasma membrane [69]. All these results exemplify how different molecular mechanisms concur to establish the pathogenicity of a variant.

The absence of exon 10 in the spliced mRNA of variants c.1535G>A and c.1537G>A would lead to an aberrant joining of exons 9 and 11 without changes in the ORF. If the mutant ClC-5 protein were expressed, it would lack 133 amino acids (amino acid residues 512 to 645), including part of α-helix O, α-helices P, Q and R, and the CBS1 domain [12]. On the other hand, skipping of exons 10 and 11 induced by variant c.1641G>T would result in the joining of exons 9 and 12 with a change in the ORF and the generation of a stop codon 13 amino acids downstream (the new sequence would be: Gly-Asp-Cys-Leu-Glu-Ser-Leu-Pro-Lys-Arg-Met-Cys-STOP). The mutant ClC-5 protein, if expressed, would lack the last 230 amino acids including part of helix O, helices P, Q, and R, and both CBS1 and CBS2 domains located at the cytoplasmic carboxy terminus of the ClC-5 protein. However, this altered mRNA would probably be degraded by NMD. 

*OCRL* variants c.1056+1G>A and c.1467-1G>A are canonical splice site variants that inactivate a donor splice site and an acceptor splice site, respectively. The consensus donor and acceptor splice sites of the pre-mRNAs contain highly conserved dinucleotides (GU, GT in the DNA, and AG) located at the beginning and the end of each intron, respectively, and that are critical for splicing [35]. The results of our functional studies with minigenes indicated that both *OCRL* variants lead to drastic changes in the respective transcripts. Variant c.1056+1G>A results in exon 11 skipping, and the joining of exons 10 and 12 does not change in the open reading frame. Therefore, the altered transcript would probably encode a non-functional OCRL1 protein lacking 39 amino acids (residues 315 to 352) in the middle of the phosphatase domain. On the other hand, variant c.1467-1G>A results in the incorporation of an exon 15 missing 20 nucleotides of the 5′ end, which involves a change in the open reading frame. The altered transcript would encode a non-functional OCRL1 protein lacking part of the phosphatase domain and the ASH and RhoGAP-like domains. In both cases, there is complete absence of the wild-type mRNA. Conversely, presumed missense variant c.2078C>T; p.(P693L) was considered as a variant with no effect on pre-mRNA splicing and, therefore, was not included in the minigene analysis. This very rare variant affects a proline residue conserved during evolution, which is located between the ASH and RhoGAP-like domains, and was predicted to affect the function of the OCRL1 protein. These two domains form a single folding module that regulates the majority of the protein–protein interactions currently described [28]. However, functional studies will be required to determine the consequences of this variant on OCRL1 activity. We have previously shown that three presumed missense variants of *OCRL* cause changes in pre-mRNA splicing. One of these variants, c.741G>T; p.(W247C), creates splicing silencer sequences (ESS) and disturbs splicing enhancer sequences (ESEs), resulting in skipping of exon 9, whereas the other two variants c.2581G>A; p.(A861T) and c.2581G>C; p.(A861P), which affect the last nucleotide of exon 23, inactivate the donor splice site, resulting in exon skipping [78]. Similarly, transcript analysis by quantitative PCR of another three *OCRL* variants, involving the last nucleotide of exons 9, 14 and 23, has shown that they affect pre-mRNA splicing [83]. 

## 5. Conclusions

We characterized the clinical and genetic characteristics of a cohort of DD patients and expanded the genetic spectrum of the disease. Using bioinformatics tools and functional analysis with a minigene system, we described the alterations of three presumed *CLCN5* missense variants and two splice-site *OCRL* variants on the splicing of pre-mRNA. Two of the *CLCN5* variants, which are close to the 5′ end of exon 10, generate overlapping ESS sites that could prevent recognition of the acceptor splice site, causing exon 10 skipping. The results of our study also showed that a presumed *CLCN5* missense variant located in exon 10 more than one hundred nucleotides away from a splice site results in skipping of both exons 10 and 11, probably through generation of ESS motifs. Furthermore, our results highlight the importance of performing functional studies to characterize the effect of canonical splice site variants on pre-mRNA splicing. Our study indicates that splicing disruption contributes to the pathogenicity of a variant in DD. The characterization of the effects of variants on pre-mRNA splicing will be very useful for the diagnosis of the disease and for the design of new therapeutic strategies.

## Figures and Tables

**Table 1:** Clinical and genetic data of patients diagnosed with Dent disease.

Patient	Age ^1^ (Years)	LMWP	HC	NL/NC	Other Symptoms	Variant	Gene	Reference
P62	15	+	+	+	−	c.952C>T; p.(R318C)	*OCRL*	[11]
P422	3	+	+	+	Hypotonia, ADD, GH deficit	c.1056+1G>A	*OCRL*	This study
P508	3.5	+	+	−	Developmental delay, congenital cataracts	c.2078C>T; p.(P693L)	*OCRL*	[62]
P652	12	+	+	−	−	c.1466G>A; p.(W489*)	*CLCN5*	[63]
P683	7	+	+	+	−	c.1560_1561delTC; p.(L521Cfs*6)	*CLCN5*	This study
P749	0.8	+	−	−	−	c.1537G>A; p.(G513R)	*CLCN5*	[64]
P801	6.5	+	+	+	−	c.1641G>T; p.(W547C)	*CLCN5*	This study
P816	19	+	+	−	−	c.976G>C; p.(G326R)	*CLCN5*	This study
P818	15	+	+	−	−	c.1558_1559insT; p.(S520Ffs*8)	*CLCN5*	[65]
P841	2.7	+	+	+	−	c.1467 − 1G>A	*OCRL*	This study
P848	10.5	+	+	−	−	c.966delC; p.(F322Lfs*37)	*CLCN5*	This study
P854	1.2	+	−	−	Hyperaminoaciduria	c.2026delA; p.(T676Lfs*2)	*CLCN5*	This study
P921	0.8	+	−	−	−	c.1600T>A; p.(Y534N)	*CLCN5*	This study

^1^ Age at diagnosis. LMWP, low-molecular-weight proteinuria; HC, hypercalciuria; NL, nephrolithiasis; NC, nephrocalcinosis; +, present; −, absent; ADD, attention-deficit disorder; GH, growth hormone.

## Data Availability

The data presented in this study are available on request from the corresponding author.

## Supplementary Materials

The following supporting information can be downloaded at: https://www.mdpi.com/article/10.3390/biomedicines11113082/s1, Table S1: Primers used in construction of minigenes and site-directed mutagenesis; Table S2: Bioinformatics analysis of novel CLCN5 and OCRL mutations identified in our study; Table S3: Bioinformatics analysis of CLCN5 missense mutations for their potential effect on pre-mRNA splicing; Table S4: Bioinformatics predictions for CLCN5 missense mutations selected from Table S3; Table S5: Generation of ESSs in CLCN5 exon 10 by mutations c.1535G>A, c.1537G>A, c. 1639T>G, c. 1639T>C and c.1641G>T, according to the bioinformatics tool HSF; Figure S1: Schematic representation of ESSs generation by exon 10 *CLCN5* mutations c.1535G>A, c.1537G>A, c. 1639T>G, c. 1639T>C and c.1641G>T, according to the bioinformatics tool HSF.
