# Supplementary material for: Characterization of pre-mRNA Splicing Defects Caused by CLCN5 and OCRL Mutations and Identification of Novel Variants Associated with Dent Disease
Source: Biomedicines. 2023 Nov 17;11(11):3082. doi: 10.3390/biomedicines11113082 (PMC10669864; doi:10.3390/biomedicines11113082)
Supplement: Supplementary file 1 [file biomedicines-11-03082-s001.zip › biomedicines-2665667-supplementary.pdf]

Table S1. Primers used in construction of minigenes and site-directed mutagenesis.

Exons	Forward primer (5′-3′)	Reverse primer (5′-3′)
CLCN5-Ex3	TTTACTCGAGGGTAAACCTCAAAGTTGTTAGTG	TATAGGATCCGGTAGAAAAACAGGAAAACACAG
CLCN5-Ex7	TTTTCTCGAGACCTGGTCAAGCATGTGAAC	TATAGGATCCGTAAAGCACCAAGTTGGGGAG
CLCN5-Ex9	ATATACTCGAGCCTTTACAGCCACTTGCTAGCATA	TTTTGGATCCTTTCCTCATCTGTATACCAGATGC
CLCN5-Ex10,11	AATCTCGAGATCGTCTCCATTGTG	ATGGGCTCTAGACTATCCACTTTCATC
OCRL-Ex11,12	TAAACTCGAGAGTTTCCACTTGACGGGGTC	ATAAGGATCCACCCTCTCCTTTGTGACCCC
OCRL-Ex15	AAACTCGAGAGTACACGGTTCTTTGGGAGC	ATTATCTAGATCTCCCTCCTGGTGTTTCCC
Mutagenesis-p.G261R	CTTTGGAGCACCTATAGGTAGAGTATTATTCAGCCTTG-	TCAAGGCTGAATAATACTCTACCTATAGGTGCTCCAAAG
Mutagenesis-p.E267A	GTGGAGTATTATTCAGCCTTGCAGAGGTAACAACTTTTCATTG	CAATGAAAAGTTGTTACCTCTGCAAGGCTGAATAATACTCCAC
Mutagenesis-p.G462S	TTATCCCTAGCATGGCTGTTAGTGCTATAGCAGGT	ACCTGCTATAGCACTAACAGCCATGCTAGGGATAA
Mutagenesis-p.M504K	CCCCCGGCCTTTATGCAAAGGTTGGGGCTG	GCTGCAGCCCTAACCATTGCATAAAGGCCG
Mutagenesis-p.G506R	CGGCCTTTATGCAATGGTTAGGGCTGCAGC	GCTGCAGCCCTAACCATTGCATAAAGGCCG
Mutagenesis-p.G506E	CCTTTATGCAATGGTTGAGGCTGCAGCCTGCTTAG	CTAAGCAGGCTGCAGCCTCAACCATTGCATAAAGG
Mutagenesis-p.G506E	CCTTTATGCAATGGTTGAGGCTGCAGCCTGCTTAG	CTAAGCAGGCTGCAGCCTCAACCATTGCATAAAGG
Mutagenesis-p.G512R	GGCTGCAGCCTGCTTACGTGAGTAGTGTTTGC	GCAAACACTACTCACGTAAGCAGGCTGCAGCC
Mutagenesis-p.G512D	TGTCTATTTCTTTGCAGATGGGGTGACTCGGATG	CATCCGAGTCACCCCATCTGCAAAGAAATAGACA
Mutagenesis-p.W547R	GAGCATCTGCCACCCGCTTGCTTGTCATGGC	GCCATGACAAGCAAGCGGGTGGCAGATGCT

Table S2. Bioinformatics analysis of novel *CLCN5* and *OCRL* mutations identified in our study.

Gene	Mutation	Distance from SS [a]	ACMG Classification (VarSome)	Effect on protein			Effect on splicing				ORF changes
				SIFT (score)	Polyphen2 (score)	MutPred2 (score)	SPANR PSI	CADD-splice [b]	Human Splicing Finder	SpliceAI [c] (score)	
CLCN5	c.1560_1561delTC (p.L521Cfs*6)	+27	Pathogenic (10 pts)	--	--	--	--	--	--	No effect (<0,2)	Yes
CLCN5	c.1641G>T (p.W547C)	+107	Likely pathogenic (8 pts)	Damaging (0.00)	Likely Damaging (1.000)	Deleterious (0.942)	↑	29.2	New donor ss	No effect (<0,2)	Yes
CLCN5	c.976G>C (p.G326R)	+172	Uncertain significance (5 pts)	Damaging (0.00)	Likely Damaging (1.000)	Deleterious (0.966)	↑	25.9	No splicing impact	No effect (<0,2)	--
CLCN5	c.966delC (p.F322Lfs*37)	+162	Pathogenic (10 pts)	--	--	--	--	--	--	No effect (<0,2)	Yes
CLCN5	c.1600T>A (p.Y534N)	+66	Likely pathogenic (6 pts)	Damaging (0.00)	Likely Damaging (1.000)	Deleterious (0.918)	↓	25.7	No splicing impact	No effect (<0,2)	--
CLCN5	c.2026delA (p.T676Lfs*2)	+93	Pathogenic (10 pts)	--	--	--	--	--	--	No effect (<0,2)	Yes
OCRL	c.1056+1G>A	+1	--	--	--	--	↓	--	Donor site inactivated	Donor site loss (0,99)	No
OCRL	c.1467-1G>A	-1	--	--	--	--	↓	--	Acceptor site inactivated	Acceptor site loss (0,99)	Yes

ESE, Exonic Splicing Enhancer; ESS, Exonic Splicing Silencer; (--) Not determined; PSI, Percentage of transcripts with the exon spliced in. ↑, higher the percentage of transcripts with the exon spliced in. ↓, lower the percentage of transcripts with the exon spliced in. n

[a] Position in relation to the acceptor (+) or donor (-) splice site (SS).

[b] CADD-splice score equal or higher than 20 indicates that the variant is damaging.

[c] SpliceAI scores range from 0 to 1 and represent the probability that the variant affects splicing. Scores ≥ 0,2 (low); ≥ 0,5 (recommended); ≥ 0,8 (high precision). Only specific scores higher than 0.2 are shown.

Table S3. Bioinformatics analysis of *CLCN5* missense mutations for their potential effect on pre-mRNA splicing.

Exon	Amino acid change	Nucleotide change	cDNA change	Codon number	Distance from splice site [a]	Coordinate	Bioinformatics tools				
							MutPredSplice [b] (score)	SPANR	Human Splicing Finder		
**3**	p.G57V	c.170G>T	GGC-GTC	57	-36	chr23:49837208G>T	SNV (0.42)	No effect	New Donor site	New ESS Site	ESE site inactivated
**3**	p.W58L	c.173G>C	TGG-TGC	58	-33	chr23:49837211G>C	SNV (0.3)	No effect	ESE Site inactivated		
**3**	p.W58C	c.174G>C	TGG-TTG	58	-32	chr23:49837212G>C	SNV (0.23)	No effect	No significant splicing motif alteration.		
**3**	p.G65R	c.193G>A	GGG-AGG	65	-13	chr23:49837231G>A	SAV (0.76)	Affects	New Acceptor site		
**4**	p.G88D	c.263G>A	GGT-GAT	88	+58	chr23:49840507G>A	SNV (0.26)	No effect	No significant splicing motif alteration.		
**4**	p.G88V	c.263G>T	GGT-GTT	88	+58	chr23:49840507G>T	SNV (0.36)	No effect	New Donor Site	New ESS Site	
**4**	p.C90W	c.270C>G	TGC-TGG	90	+65	chr23:49840514C>G	SNV (0.13)	No effect	No significant splicing motif alteration.		
**6**	p.I176N	c.527T>A	ATC-AAC	176	+11	chr23:49846308T>A	SNV (0.32)	No effect	No significant splicing motif alteration.		
**6**	p.G179D	c.536G>A	GGT-GAT	179	+20	chr23:49846317G>A	SNV (0.44)	No effect	New Donor Site	ESE Site inactivated	
**6**	p.C219R	c.655T>C	TGC-CGC	219	-69	chr23:49846436T>C	SNV (0.18)	No effect	ESE Site Broken		
**6**	p.C221R	c.661T>C	TGT-CGT	221	-63	chr23:49846442T>C	SNV (0.21)	No effect	ESE Site inactivated		
**6**	p.L225P	c.674T>C	CTG-CCG	225	-50	chr23:49846455T>C	SNV (0.21)	No effect	ESE Site inactivated		
**6**	p.K231I	c.692A>T	AAA-ATA	231	-32	chr23:49846473A>T	SNV (0.47)	No effect	ESE Site inactivated		
**6**	pR239P	c.716G>C	CGC-CCC	239	-8	chr23:49846497G>C	SNV (0.47)	No effect	ESE Site inactivated		

Table S3. Continuation

Exon	Amino acid change	Nucleotide change	cDNA change	Codon number	Distance from splice site [a]	Coordinate	Bioinformatics tools				
							MutPredSplice [b] (score)	SPANR	Human Splicing Finder		
**7**	p.S244L	c.731C>T	TCG-TTG	244	+8	chr23:49850644C>T	SNV (0.34)	Affects	New ESS Site		
**7**	p.G250R	c.748G>C	GGT-CGT	250	+25	chr23:49850661G>C	SNV (0.33)	No effect	ESE Site inactivated		
**7**	p.G260V	c.779G>T	GGT-GTT	260	+26	chr23:49850692G>T	SNV (0.34)	No effect	New Donor Site	New ESS Site	ESE Site inactivated
**7**	p.G261R	c.781G>A	GGA-AGA	261	-24	chr23:49850694G>A	SAV (0.93)	Affects	New ESS Site	ESE Site inactivated	
**7**	p.L263F	c.789A>T	TTA-TTT	263	-16	chr23:49850702A>T	SNV (0.37)	Affects	No significant splicing motif alteration.		
**7**	p.S265R	c.793A>C	AGC-CGC	265	-12	chr23:49850706A>C	SNV (0.47)	No effect	ESE Site inactivated		
**7**	p.L266V	c.796C>G	CTT-GTT	266	-9	chr23:49850709C>G	SNV (0.36)	No effect	ESE Site inactivated		
**7**	p.Q267A	c.800A>C	GAA-GCA	267	-5	chr23:49850713A>C	SNV (0.44)	Affects	New ESS Site	ESE Site inactivated	
**7**	p.Q267D	c.801A>C	GAA-GAC	267	-4	chr23:49850714A>C	SNV (0.49)	No effect	ESE Site inactivated		
**8**	p.S270R	c.808A>C	AGC-CGC	270	+4	chr23:49850988A>C	SAV (0.63)	No effect	No significant splicing motif alteration.		
**8**	p.S270G	c.808A>G	AGC-GGC	270	+4	chr23:49850988A>G	SAV (0.66)	No effect	No significant splicing motif alteration.		
**8**	p.S270R	c.810C>G	AGC-AGG	270	+6	chr23:49850990C>G	SNV (0.49)	No effect	ESE Site inactivated		
**8**	p.Y272N	c.814T>A	TAT-AAT	272	+10	chr23:49850994T>A	SNV (0.29)	No effect	No significant splicing motif alteration.		
**8**	p.Y272C	c.815A>G	TAT-TGT	272	+11	chr23:49850995A>G	SNV (0.35)	No effect	No significant splicing motif alteration.		
**8**	p.F273L	c.817T>C	TTT-CTT	273	+13	chr23:49850997T>C	SNV (0.27)	No effect	No significant splicing motif alteration.		

Table S3. Continuation

Exon	Amino acid change	Nucleotide change	cDNA change	Codon number	Distance from splice site [a]	Coordinate	Bioinformatics tools				
							MutPredSplice [b] (score)	SPANR	Human Splicing Finder		
**8**	p.L278W	c.833T>G	TTG-TGG	278	+29	chr23:49851013T>G	SNV (0.30)	No effect	New Donor Site	New ESS Site	ESE Site inactivated
**8**	p.L278S	c.833T>C	TTG-TCG	278	+29	chr23:49851013T>C	SNV (0.14)	No effect	ESE Site inactivated		
**8**	p.L278F	c.834G>C	TTG-TTC	278	+30	chr23:49851014G>C	SNV (0.13)	No effect	ESE Site inactivated		
**8**	p.R280P	c.839G>C	CGT-CCT	280	+35	chr23:49851019G>C	SNV (0.14)	No effect	New ESS Site		
**9**	p.G462S	c.1384G>A	GGT-AGT	462	+37	chr23:49853391G>A	SAV (0.62)	No effect	New Acceptor site / Donor Site		
**9**	p.G462D	c.1385G>A	GGT-GAT	462	+38	chr23:49853392G>A	SNV (0.39)	No effect	No significant splicing motif alteration		
**9**	p.G462V	c.1385G>T	GGT-GTT	462	+38	chr23:49853392G>T	SNV (0.25)	No effect	New ESS Site		
**9**	p.G466R	c.1396G>C	GGT-CGT	466	+49	chr23:49853403G>C	SNV (0.24)	No effect	No significant splicing motif alteration		
**9**	p.G466D	c.1397G>A	GGT-GAT	466	+50	chr23:49853404G>A	SNV (0.20)	No effect	No significant splicing motif alteration		
**9**	p.L468P	c.1403T>C	CTT-CCT	468	+56	chr23:49853410T>C	SNV (0.12)	No effect	No significant splicing motif alteration		
**9**	p.L469P	c.1406T>C	CTA-CCA	469	+59	chr23:49853413T>C	SNV (0.12)	No effect	New ESS Site		
**9**	p.G470R	c.1408G>A	GGA-AGA	470	+61	chr23:49853415G>A	SNV (0.43)	No effect	No significant splicing motif alteration		
**9**	p.Y502C	c.1505A>G	TAT-TGT	502	-30	chr23:49853512A>G	SNV (0.18)	No effect	ESE Site inactivated		
**9**	p.M504K	c.1511T>A	ATG-AAG	504	-24	chr23:49853518T>A	SAV (0.70)	No effect	New Acceptor site / Donor Site		
**9**	p.V505G	c.1514T>G	GTT-GGT	505	-21	chr23:49853521T>G	SNV (0.38)	No effect	New Donor Site		
**9**	p.G506R	c.1516G>A	GGG-AGG	506	-19	chr23:49853523G>A	SAV (0.67)	No effect	New Acceptor site / Donor Site		

Table S3. Continuation

Exon	Amino acid change	Nucleotide change	cDNA change	Codon number	Distance from splice site [a]	Coordinate	Bioinformatics tools		
							MutPredSplice [b] (score)	SPANR	Human Splicing Finder
**9**	p.G506Q	c.1517G>A	GGG-GAG	506	-18	chr23:49853524G>A	SNV (0.24)	No effect	New acceptor Site
**9**	p.G512R	c.1534G>C	GGT-CGT	512	-1	chr23:49853541G>C	SAV (0.80)	No effect	Donor Site inactivated
**10**	p.G512D	c.1535G>A	GGT-GAT	512	+1	chr23:49854773G>A	SAV (0.81)	No effect	No significant splicing motif alteration
**10**	p.G513R	c.1537G>A	GGG-AGG	513	+3	chr23:49854775G>A	SAV (0.80)	No effect	New Acceptor site
**10**	p.G513Q	c.1538G>A	GGG-GAG	513	+4	chr23:49854776G>A	SAV (0.84)	No effect	New Acceptor site / New Donor site
**10**	p.R516W	c.1546C>T	CGG-TGG	516	12	chr23:49854784C>T	SNV (0.26)	No effect	New ESS Site
**10**	p.R516Q	c.1547G>A	CGG-CAG	516	13	chr23:49854785G>A	SNV (0.32)	No effect	New ESS Site
**10**	p.T518A	c.1552A>G	ACT-GCT	518	18	chr23:49854790A>G	SNV (0.34)	No effect	New ESS Site<br>ESE Site inactivated
**10**	p.V519D	c.1556T>A	GTT-GAT	519	22	chr23:49854794T>A	SNV (0.26)	No effect	No significant splicing motif alteration
**10**	p.S520P	c.1558T>C	TCT-CCT	520	24	chr23:49854796T>C	SNV (0.21)	No effect	No significant splicing motif alteration
**10**	p.L521F	c.1561C>T	CTT-TTT	521	27	chr23:49854799C>T	SNV (0.37)	No effect	No significant splicing motif alteration
**10**	p.I524K	c.1571T>A	ATA-AAA	524	37	chr23:49854809T>A	SNV (0.17)	No effect	No significant splicing motif alteration
**10**	p.Q527D	c.1581A>T	GAA-GAT	527	47	chr23:49854819A>T	SNV (0.20)	No effect	ESE Site inactivated
**10**	p.W547R	c.1639T>C	TGG-CGG	547	105	chr23:49854877T>C	SAV (0.81)	No effect	No significant splicing motif alteration
**11**	p.T657S	c.1970C>G	ACT-AGT	657	37	chr23:49855363C>G	SNV (0.19)	No effect	No significant splicing motif alteration

Table S3. Continuation

Exon	Amino acid change	Nucleotide change	cDNA change	Codon number	Distance from splice site [a]	Coordinate	Bioinformatics tools		
							MutPredSplice [b] (score)	SPANR	Human Splicing Finder
**11**	p.F703S	c.2108T>C	TCC-CCC	703	-43	chr23:49855501T>C	SNV (0.11)	No effect	No significant splicing motif alteration
**11**	p.L706P	c.2117T>C	CTG-CCG	706	-34	chr23:49855510T>C	SNV (0.33)	No effect	ESE Site inactivated
**11**	p.C711W	c.2133C>G	TGC-TGG	711	-18	chr23:49855526C>G	SNV (0.17)	No effect	New ESS Site<br>ESE Site inactivated

Mutations were obtained from reference 38. ESE, Exonic Splicing Enhancer; ESS, Exonic Splicing Silencer; SNV, Splice Neutral Variant; SAV, Splice Affecting Variant

a. Position in relation to the acceptor (+) or donor (-) splice site.

b. The general score of MutPredSplice ranges from 0.0 and 1.0, with a higher score indicating a greater propensity to be pathogenic.

Table S4. Bioinformatics predictions for *CLCN5* missense mutations selected from Table S3.

Exon	Mutation	Distance from SS [a]	ACMG classification (VarSome)	Effect on protein			Effect on splicing					
				SIFT (score)	Polyphen2 (score)	MutPred2 (score)	MutPred Splice (score) [b]	SPANR PSI	CADD-splice [c]	Human Splicing Finder	SpliceAI (score)[d]	ORF changes
**3**	c.193G>A (p.G65R)	-13	Uncertain significance (5 pts)	Damaging (0.00)	Probably damaging (1.00)	Deleterious (0.950)	SAV (0.76)	↓	31	New acceptor ss	No effect (<0,2)	No
**7**	c.731C>T (p.S244L)	+8	Likely pathogenic (9 pts)	Damaging (0.00)	Probably damaging (0.986)	Deleterious (0.899)	SNV (0.34)	↓	26.9	New ESS or ESE site New acceptor or donor ss	0,00	No
**7**	c.781G>A (p.G261R)	-24	Likely pathogenic (7 pts)	Damaging (0.00)	Probably damaging (1.00)	Deleterious (0.958)	SAV (0.93)	↓	33	New ESS or ESE site	Donor gain (0.55)	No
**7**	c.800A>C (p.E267A)	-5	Likely pathogenic (8 pts)	Damaging (0.00)	Probably damaging (0.987)	Deleterious (0.910)	SNV (0.44)	↓	27.9	New ESS or ESE and ESS or ESE site inactivated	No effect (<0,2)	No
**9**	c.1384G>A (p.G462S)	+37	Likely pathogenic (9 pts)	Damaging (0.00)	Probably damaging (0.999)	Deleterious (0.950)	SAV (0.62)	↓	27.2	New acceptor/ donor ss	No effect (<0,2)	No
**9**	c.1511T>A (p.M504K)	-24	Likely pathogenic (7 pts)	Damaging (0.00)	Probably damaging (0.948)	Deleterious (0.951)	SAV (0.7)	↑	26.1	New acceptor/ donor ss	No effect (<0,2)	No
**9**	c.1516G>A (p.G506R)	-19	Likely pathogenic (7 pts)	Damaging (0.01)	Probably damaging (1.00)	Deleterious (0.960)	SAV (0.67)	↑	29.9	New acceptor/ donor ss	No effect (<0,2)	No
**9**	c.1517G>A (p.G506E)	-18	Likely pathogenic (7 pts)	Damaging (0.00)	Probably damaging (0.999)	Deleterious (0.960)	SNV (0.24)	↑	27.6	New acceptor ss	0,00	No
**9**	c.1534G>C (p.G512R)	-2	Pathogenic (10 pts)	Damaging (0.01)	Probably damaging (1.00)	Deleterious (0.928)	SAV (0.8)	↑	33	Donor site inactivated	No effect (<0,2)	No

**10**	c.1535G>A (p.G512D)	+1	Pathogenic (13 pts)	Damaging (0.02)	Probably damaging (0.999)	Deleterious (0.931)	SAV (0.81)	↑	32	No splicing impact	No effect (<0,2)	No
**10**	c.1537G>A (p.G513R)	+3	Pathogenic (14 pts)	Damaging (0.02)	Likely damaging (0.714)	Deleterious (0.911)	SAV (0.80)	--	29.5	New acceptor ss	Acceptor gain (0.22)	No
**10**	c.1639T>C (p.W547R)	+105	Pathogenic (11 pts)	Damaging (0.00)	Probably damaging (0.998)	Deleterious (0.932)	SAV (0.81)	--	27.6	No splicing impact	0,00	No

ESE, Exonic Splicing Enhancer; ESS, Exonic Splicing Silencer; (--) Not determined; SAV, Splicing Affecting Variant; SNV, Splicing Neutral Variant; PSI, Percentage of transcripts with the exon spliced in; PSI, Percentage of transcripts with the exon spliced in. ↑, higher the percentage of transcripts with the exon spliced in. ↓, lower the percentage of transcripts with the exon spliced in.

a. Position in relation to the acceptor (+) or donor (-) splice site (SS).

b. The general score of MutPredSplice ranges from 0.0 and 1.0, with a higher score indicating a greater propensity to be pathogenic.

c. CADD-splice score equal or higher than 20 indicates that the variant is damaging.

d. SpliceAI scores range from 0 to 1 and represent the probability that the variant affects splicing. Scores ≥ 0,2 (low); ≥ 0,5 (recommended); ≥ 0,8 (high precision). Only specific scores higher than 0.2 are shown.

Table S5. Generation of ESSs in *CLCN5* exon 10 by mutations c.1535G>A, c.1537G>A, c. 1639T>G, c. 1639T>C and c.1641G>T, according to the bioinformatics tool HSF.

Position	ESS	WT sequence	mt c.1535G>A sequence	WT CV	c.1535G>A CV	ΔCV
1	Sironi_motif1	--	GCAGaTGG	NP	62,20	Novo
1	Sironi_motif2	--	AGaTGGG	NP	82,65	Novo
1	ESS_hnRNPA1	--	GaTGGG	NP	66,79	Novo
1	Sironi_motif2	--	GaTGGGG	NP	80,51	Novo

Position	ESS	WT sequence	mt c.1537G>A sequence	WT CV	c.1537G>A CV	ΔCV
1	Sironi_motif2	--	GTaGGGT	NP	64,4	Novo
2	Sironi_motif2	TGGGGTG	--	71,04	NP	None
2	ESS_hnRNPA1	--	TaGGGT	NP	99,41	Novo

Position	ESS	WT sequence	mt c. 1639T>G sequence	WT CV	c.1639T>G CV	ΔCV
100	Sironi_motif2	--	GCAAGgG	NP	62,31	Novo
101	ESS_hnRNPA1	--	CAAGgG	NP	65,13	Novo
101	Sironi_motif2	--	CAAGgGG	NP	70,66	Novo
101	Sironi_motif1	CAAGTGGG	CAAGgGGG	75,17	75,17	0
102	ESS_hnRNPA1	AAGTGG	AAGgGG	67,03	82,27	-15,24
102	Sironi_motif2	--	AAGgGGG	NP	64,21	Novo
102	Sironi_motif1	--	AAGgGGGT	NP	61,16	Novo
103	Sironi_motif2	AGTGGGT	AGgGGGT	81,60	69,06	12,54
104	Sironi_motif2	--	GgGGGTG	NP	67,96	Novo
105	Sironi_motif2	TGGGTGG	gGGGTGG	71,04	67,96	3,08

Position	ESS	WT sequence	mt 1639T>C sequence	WT CV	1639T>C CV	ΔCV
101	Sironi_motif1	CAAGTGGG	CAAGcGGG	75,17	75,17	0
102	ESS_hnRNPA1	AAGTGG	AAGcGG	67,03	67,03	0
103	Sironi_motif2	AGTGGGT	AGcGGGT	81,60	69,06	12,54
105	Sironi_motif2	TGGGTGG	cGGGTGG	71,04	61,17	9,87

Position	ESS	WT sequence	mt c.1641G>T sequence	WT CV	c.1641G>T CV	ΔCV
101	Sironi_motif1	CAAGTGGG	CAAGTGtG	75,17	61,64	13,54
102	ESS_hnRNPA1	AAGTGG	--	67,03	NP	None
103	Sironi_motif2	AGTGGGT	AGTGtGT	81,60	64,69	-1,87
105	Sironi_motif2	TGGGTGG	TGtGTGG	71,04	83,58	-16,19
106	Sironi_motif2	GGGTGGC	--	66,88	NP	None
107	Sironi_motif2	GGTGGCA	tGTGGCA	69,67	72,75	-12,54

WT, exon 10 wilt-type motif sequence; mt, exon 10 mutated motif sequence; mutant nucleotides are in small letters; --, motif no observed; CV, consensus values from Human Splicing Finder (the higher the score, the higher the confidence that the corresponding trans-acting factors recognizes the site); ΔCV, WT CV – Mutant CV (If differences are negative, the ESS confidence is higher in mutation); NP, CV not present; None, no CV differences; *Novo*, ESS motifs only observed in mutated sequence

c.1535G>A

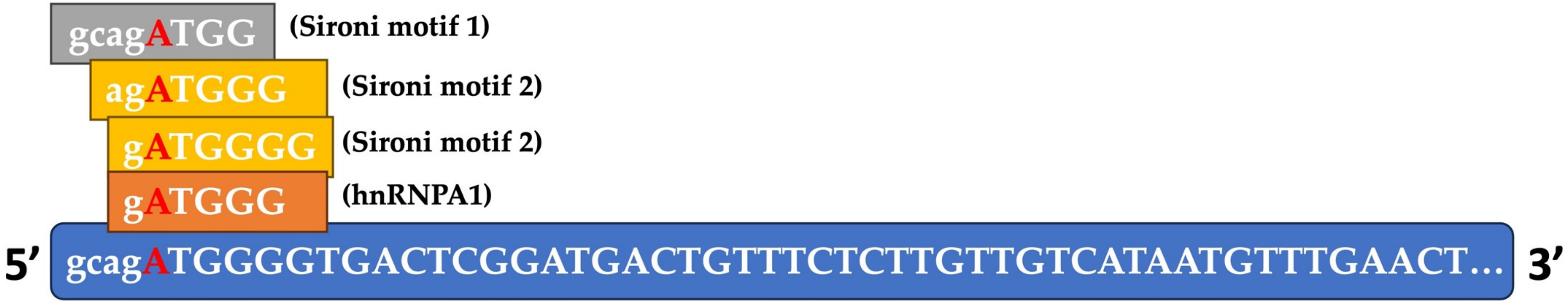

c.1537G>A

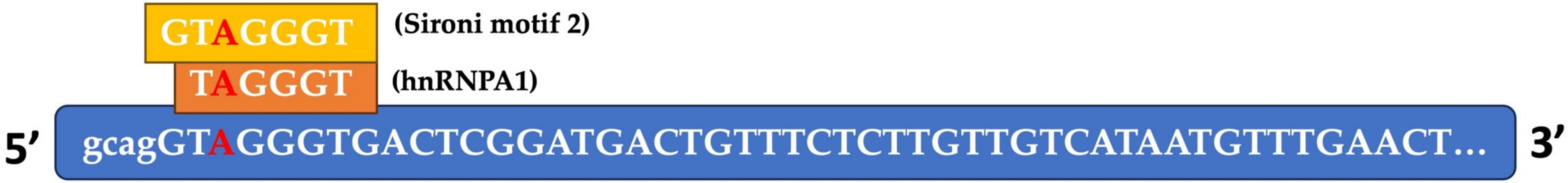

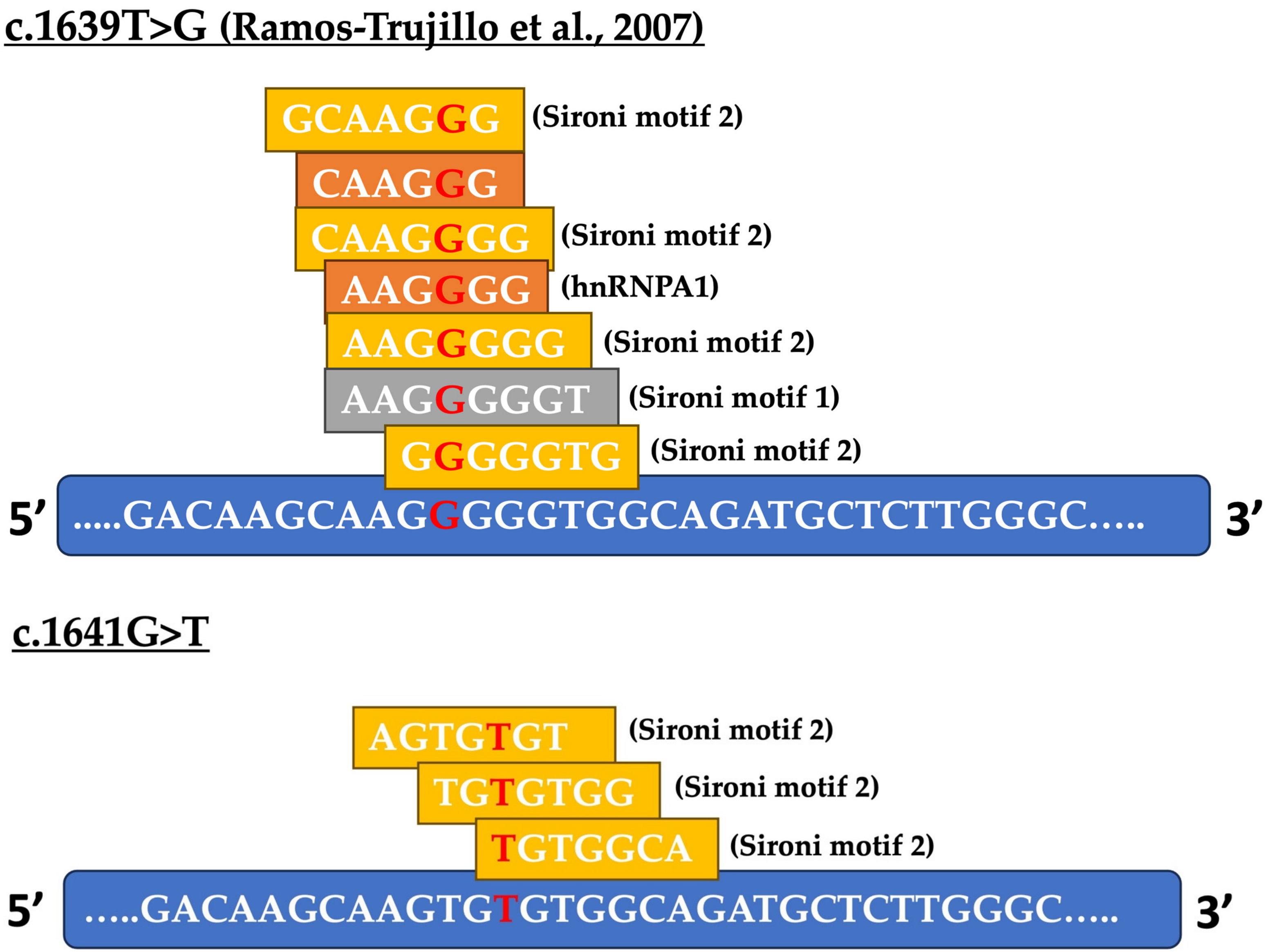

Figure S1. Schematic representation of ESSs generation by exón 10 *CLCN5* mutations c.1535G>A, c.1537G>A, c. 1639T>G, c. 1639T>C and c.1641G>T, according to the bioinformatics tool HSF.

Intron sequences are in small letters and exon sequences in capital letters; ESSs showed are novo sequences or with negative ΔCV. Sironi motif 1, are marked with a gray box; Sironi motif 2, are marked with a yellow box; hnRNPA1 motif are marked with orange box.
